# Influences of different parameters on selected properties of gears for robot-like systems

**DOI:** 10.3389/frobt.2024.1414238

**Published:** 2024-08-20

**Authors:** Florian Oberneder, Stefan Landler, Michael Otto, Birgit Vogel-Heuser, Markus Zimmermann, Karsten Stahl

**Affiliations:** ^1^ Institute for Machine Elements, Gear Research Center (FZG), Technical University of Munich, Munich, Germany; ^2^ Institute for Automation and Information Systems (AIS), Technical University of Munich, Munich, Germany; ^3^ Laboratory for Product Development and Lightweight Design (LPL), Technical University of Munich, Munich, Germany

**Keywords:** robot drive system, robot gears, properties, efficiency, stiffness, harmonic drive, cycloidal drive, planetary drive

## Abstract

For a drive unit for axes of robots and robot-like systems (RLS) usually a motor-gearbox arrangement is chosen due to its high-power density. The combination of a high-ratio gearbox and a high-speed electric motor ensures a very compact and efficient design of the drive train. The transmission properties primarily determine the properties of the axes and the whole robot system. Robots and RLS use various types of high-ratio precision gearboxes based on different operating principles. Due to the different operating principles, it is difficult to describe comparable properties across all different types. In addition, there are many influences on the properties which significantly determine their shapes and values. These influencing parameters are insufficiently documented and are often poorly accessible for profound comparability and further consideration. In this paper, an overview of the properties of robot gearboxes is given. Based on these properties, different robot gearboxes can be systematically evaluated and compared to one another. The properties are influenced by various design, operating or manufacturing factors such as the gearbox size, the operating torque and speed or the manufacturing process. In a further step, these influences on the most relevant properties, efficiency and stiffness, are determined and systematically evaluated. This evaluation is based on the specification data of various robot gearbox manufacturers. The properties efficiency and stiffness show a dependency on the gearbox size, the operating torque, speed as well as the ambient temperature and on the transmission ratio. The shown procedure can also be adapted to other properties.

## 1 Introduction

ISO 8373 (ISO, 2021) defines an industrial robot as an “automatically controlled, reprogrammable multipurpose manipulator […] for use in automation applications in an industrial environment.” Based on this definition a large production plant is an industrial robot as well as a multi-axes articulated robot (Vogel-Heuser et al., 2020). Despite the very different areas of application, there are many similarities between these two examples. The drive units and especially the transmissions are thus comparable and require similar functionalities such as the synchronized control of the axes. Therefore, these applications can be referred to as robot-like systems (RLS). RLS can cover both large production plants in the high torque range and SCARA (Selective Compliance Assembly Robot Arm) in the low torque range (Volpert et al., 2024). An example of an RLS could be a production machine like the continuous press with co-rotating conveying belts, see (Vogel-Heuser et al., 2024).

Industrial robots operate in a wide range of applications. According to the International Federation of Robotics (Müller, 2022) the three main areas of applications are handling, welding and assembling tasks. Pick-and-Place operations are typical examples of handling operations. This task can be described in three steps: Pick up an object, transport the object and place the object at a specific position. Such robots are for example, used in the food industry for the packaging of food (Sharma and Harada, 2023), in the electrical industry for the assembly of printed circuit boards (Hu et al., 2022) and in mechanical engineering industry for loading and unloading milling machines (Brecher and Nittinger, 2017). Although the workflow for these three examples is always the same, the requirements for the tasks are very different. The pick-and-place task in the food industry requires a high acceleration over a short distance. Therefore, the inertia of the robot system should be low. By contrast, the assembly of printed circuit boards requires very high positioning accuracy. In this case, the objects are significantly smaller and the mass is irrelevant. When loading and unloading heavy production machines (e.g., milling machines), the mass of the objects is more important. In order to minimize deflection, a stiff connection of the robot system must be ensured.

All these examples show that the requirements on robot systems depend on the specific task. Special robot designs with different advantages and disadvantages have been developed for the various applications in order to meet the specific requirements, e.g., on payload, workspace and accuracy as best as possible (Siciliano, 2016). Examples for these special robot designs are the Delta robot, the SCARA robot or the articulated robot, which can be classified according to their number of axes and degrees of freedom (DOF) (Siciliano, 2016; Mareczek, 2020). A Delta robot is a three DOF robot, which can operate at high speeds and accelerations thanks to its low inertia. They are suitable e.g., for packaging tasks in the food industry. On the contrary, a SCARA robot has one more degree of freedom with a good repeatability and high velocity performance (e.g., for assembling tasks). In the case that more DOFs and larger workspaces are required, articulated robots with six degrees of freedom are usually used. To compare the different robot designs to one another, ISO 9283 (ISO, 1998) defines performance properties (Weber, 2005) and their test procedures for the whole robot system. Depending on the specific task and therefore the specific design, the relevant properties can vary on the level of the robot system. Comparing specific designs is often barely feasible because significant parameters describing the advantages and disadvantages of the different systems are often poorly accessible. This lack of information results from the fact that no sufficient method for obtaining data from suppliers’ documentation exists in literature. A first approach toward systematic data extraction can be found in (Vogel-Heuser et al., 2024).

The overall system of the RLS can be divided into different sub-systems. For the articulated robot, for example, the overall system would be the drive system and the manipulator, the drive sub-system consists, among others, of the components transmission and motor. Most system performance measures, such as speed or accuracy, are significantly influenced by components (Rosenbauer, 1994). To ensure that design goals related to the robot system performance are reached, component properties can be assigned to design targets based on so-called solution spaces (Zimmermann and Hoessle, 2013; Zimmermann et al., 2017). For a compact design of the drive system, an electrical motor (e.g., step motor) is usually combined with a high ratio transmission. Similar to the different robot designs there are also different gearbox types with specific advantages and disadvantages. The main types of gears used in industrial robots are strain wave drives, cycloidal drives and planetary gear drives (Pham and Ahn, 2018). All of them have high transmission ratios in a minimal installation space in common and are explained in Section 2.1 in more detail.

Robot movements of any task are characterized by permanent acceleration and braking processes (Ziegler et al., 2023). In this unsteady mode of operation, the load-deformation behavior including gear backlash plays a significant role. At the same time, friction processes have to be taken into account when dimensioning the driveline. Friction is particularly high in the partial load range and can therefore be decisive. The energy consumption of robotic systems is therefore becoming increasingly important (Kashiri et al., 2018). For this reason, Siciliano (2016), Rosenbauer (1994) and Gerstmann (1991) name the stiffness and efficiency as the two most relevant properties of robot gears. The specifications of the gearbox properties are influenced by different parameters. These can be operating parameters like speed, torque and temperature (Zhou et al., 2019) or manufacturing and design parameters like the manufacturing process or the material of the gearbox (Hasl et al., 2018). Because of different working principles (see Section 2.1), a general description of these influences on the properties is difficult and not sufficiently researched.

In this paper, the properties of the different types of gears for RLS are summarized. Similarly, an overview of possible influences on the properties is shown. Based on this overview, the main influences are illustrated in detail on the example of the two most relevant properties stiffness and efficiency.

## 2 State of the art

### 2.1 Drive systems for robots and RLS

**FIGURE 1:**
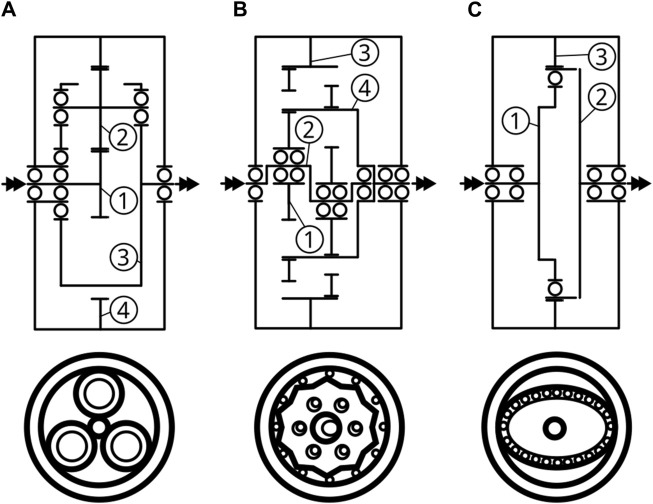
Schemes of coaxial drives for robots or RLS. **(A)** Planetary gear drive. **(B)** Cycloidal drive. **(C)** Strain wave drive.

In most of the cases, the joints of industrial robots are actuated by an electromagnetic motor (Siciliano, 2016). To achieve a compact design, the motor is usually combined with a high-ratio transmission (Hollerbach et al., 1992). The most commonly used transmissions for robot drives are planetary gear drives (PG), cycloidal drives (CY) and strain wave drives (SW) (Sensinger and Lipsey, 2012; Pham and Ahn, 2018; Lopez Garcia et al., 2020). Although these three categories are based on different working principles, they can still be classified in the same overall group as a coaxial epicyclic gear (Rosenbauer, 1994). For a better understanding and description of the principle, functional schemes are used to describe the working structure (Tsai, 2001; Pennestrí and Valentini, 2002; Pham and Ahn, 2018; Lopez Garcia et al., 2020; Landler et al., 2023). The schemes of the three different types are shown in the top of Figure 1. In addition, a symbolic representation of the front view of the three drives is shown at the bottom of the Figure.

A simple epicyclic gear consists of three central elements that are arranged coaxially. In addition, there are eccentrically arranged elements rotating around the central axis and their own axis. The different central elements can be used either as input or output of the system (Müller, 1998; Arnaudov and Karaivanov, 2019). For robot drives, one of the central shafts is fixed, one is used as the input and one as the output of the system. Depending on the selected arrangement of the central elements, six different transmission ratios are possible (Gravagno et al., 2021). In the following section, only the constellation with the highest transmission ratio is mentioned.

In the case of a simple planetary drive (see Figure 1A), the central elements are the sun gear (1), the carrier (3) and the ring gear (4). For a high transmission ratio the sun gear (1) is the input of the system, which meshes with eccentrically arranged planetary gears (2). In order to achieve an even load distribution, a minimum of three planetary gears are used, which are mounted in the second central element – the carrier. The planetary gears mesh also with the fixed, internally toothed ring gear and thus generate the rotation of the carrier as output of the system (Müller, 1998; Verein Deutscher Ingenieure e.V., 2012). The transmission ratio of simple planetary drives is limited due to constructive boundaries (Müller, 1998). To achieve even higher transmission ratios, either several simple planetary gear trains are combined together, or special configurations with a compact design (e.g., Wolfrom planetary gears (Mihailidis and Nerantzis, 2013; Höhn et al., 2014)) are used (Lopez Garcia et al., 2020). Another special feature in robot applications is the requirement of zero backlash, which is not standard in regular planetary gear units. Tönshoff et al. (1990) propose therefore options in axial, radial or tangential direction to reduce the backlash in planetary gears for robot applications. The use of conical-shaped gears, as mentioned in (Höhn et al., 2011), is an example of a reduction in the axial direction.

The cycloidal drive is a special design of an epicyclic gear (see Figure 1B). The cycloidal disc (1), as the eccentrical element, is mounted on an eccentric cam of the input shaft (2). The disc meshes with the pins of a fixed ring gear (3) and transfers the power to the output rollers of the output shaft (4), which meshes with the inner holes of the disc. For mass balancing, more than one cycloidal disc is used and arranged symmetrically in the system (Maccioni et al., 2020). Since the cycloidal toothing of the cycloidal disc is connected to the pins of the ring gear with a large number of contacts, cycloidal gears are characterized by high stiffness. Hsieh/Fuentes-Aznar (Hsieh and Fuentes-Aznar, 2019) investigated the influences of design parameters on cycloidal drives. The number of output rollers greatly influences the performance of the drive system. An alternative concept with higher performance is achieved by a combination of a planetary gear and a cycloidal drive. It is called the RV reducer (Han and Guo, 2016). Due to the load distribution, this concept results in a better overall performance.

Another special epicyclic drive is the strain wave drive, which is better known under the manufacturer name Harmonic Drive. The three central elements (see Figure 1C) are the wave generator (1), the flexspline (2) and the circular spline (3). The wave generator is connected to the input of the system and has an elliptical shape. A flexible roller bearing is mounted on this shape and connects the wave generator with a flexible steel ring (flexspline), which is also forced into the elliptical form of the wave generator. Due to this elliptical shape, the external teeth of the flexspline mesh with the teeth of the internally toothed, fixed ring gear (circular spline) on two oppositely located contact points. The flexspline has a slightly smaller number of teeth than the circular spline (Musser, 1955). This small difference in the number of teeth causes a high transmission ratio and a high stiffness because many teeth are engaged at the same time.

Recent investigations deal with alternative materials for robot gears. Paetzold (2004), Blagojevic et al. (2017), Hasl et al. (2018) therefore demonstrate the possibility of using plastic gears for the three gearbox types. Plastic gears have advantages in the design process, the manufacturing process and the NVH (Noise-Vibration-Harshness) behavior (Biernacki, 2014).

Changing the material from steel to plastic, changing the manufacturing process or even changing the type of gear in a RLS may cause significant changes in the properties of the drive system. Therefore a simple replacement of the transmission in a robot system is challenging (Vogel-Heuser et al., 2020).

### 2.2 Properties and their influences

**FIGURE 2:**
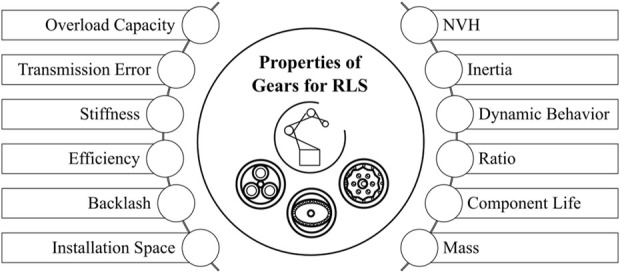
Properties of gears in RLS.

Requirements on the overall robot system can be used to derive requirements on the transmission properties. Rosenbauer (1994), Gerstmann (1991), Mayr (1989), Slatter and Mackrell (1994) describe different performance criteria for gearboxes of industrial robots. Pham and Ahn (2018), Lopez Garcia et al. (2020) use such performance criteria to compare the main types of drives. According to this literature, the main properties of gears for robot drives can be summarized as following (see Figure 2):

According to Siciliano (2016), Rosenbauer (1994), Gerstmann (1991), the two most relevant properties for gears in RLS are the stiffness and the efficiency.

Due to energy consumption and sustainability requirements, the efficiency is a very important characteristic for robot gears. It is defined as the quotient of the output power *P*_*Out*_ to input power *P*_*In*_, wherein the output power results from the subtraction of the input power minus the total power loss *P*_*V*_:

$$\eta =\frac{P_{Out}}{P_{In}}=\frac{P_{In}-P_{V}}{P_{In}}$$ (1)

The efficiency of the different gear types for RLS is the topic of different investigations. Farrell et al. (2018) analyze the efficiency of cycloidal drives experimentally and compare it to strain wave drives. The efficiency of the cycloidal drive is slightly higher than the efficiency of a comparable strain wave drive and is mainly dependent on the torque of the system. Gravagno et al. (2021) define a model to calculate the efficiency of a strain wave drive and compare the numerical solution with experimental data from the manufacturer. The model is also capable to map the effects of torque, temperature and speed on efficiency. For planetary gear drives, different calculation approaches are established. ISO/TR 14179-1 (ISO, 2001) therefore divides the power losses of cylindrical gears into load-dependent *P*_*VP*_ and load-independent losses *P*_*V0*_:

$$P_{V}=P_{VP}+P_{V0}$$ (2)

The load-dependent losses itself can be separated into gear power losses *P*_*VZP*_ and into bearing power losses *P*_*VLP*_, the load-independent losses into load-independent gear mesh losses *P*_*VZ0*_, load-independent bearing losses *P*_*VL0*_, sealing losses *P*_*VD*_ and losses of other components *P*_*VX*_ (e.g., oil pump losses) (Kurth, 2012).

Therefore, Equation 2 changes to:

$$P_{V}=P_{VZP}+P_{VLP}+P_{VZ0}+P_{VL0}+P_{VD}+P_{VX}$$ (3)

Complex calculation approaches can be found for each individual share of Equation 3, for example, in ISO/TR 14179-1 (ISO, 2001). All of these calculation methods demonstrate different geometric, operational and material-related influences. For example, the calculation method according to Niemann and Winter (2003) for the mesh power losses *P*_*VZP*_ is dependent on the geometric shape of the gear like tip contact ratio, helix angle or the number of teeth, on operating conditions like the sliding speed or contact force, and also on material parameters like friction coefficient or the dynamic oil viscosity.

**FIGURE 3:**
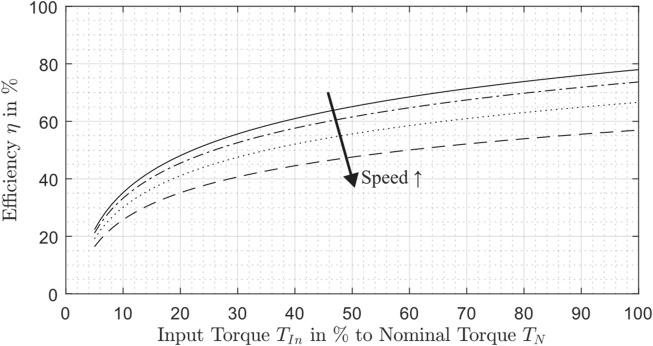
Example behavior of the efficiency in dependency on torque and speed.

A gear manufacturer of gears for RLS typically provides only information about the overall efficiency value dependent on the operating point. Figure 3 shows an example of the efficiency curve in dependency on torque and speed.

With rising torque, the efficiency also increases, whereas the influence of speed on the efficiency shows the opposite effect. Schafer et al. (2005) demonstrate such effects with experimental measurements on strain wave drives.

Next to the efficiency the load-deformation behavior of gears for RLS is of interest. The corresponding characteristic for the evaluation is the stiffness. The value can be calculated in a general way as the ratio of the applied torque to the torsional deformation (twist angle) (see Equation 4):

$$c_{\varphi }=\frac{∆T}{∆\varphi }$$ (4)

The stiffness characteristic of robot gears is composed of the deformation of different parts in the gearbox (e.g., housing, bearing, gear, …) (Landler et al., 2023). The gear deformation is one of the most important influence factor on the overall stiffness characteristic and is mainly determined by the bending deformation, shear deformation and deformation due to Hertzian contact (Linke et al., 2016). There are specific investigations on the stiffness performance for the individual types of robot gears. ISO 6336-1 (ISO, 2019) provides general calculation methods valid for involute gears. Hochrein et al. (2022) describe a fast method to calculate the tooth deflection for spur gears based on the approach of Weber and Banaschek (1953).

**FIGURE 4:**
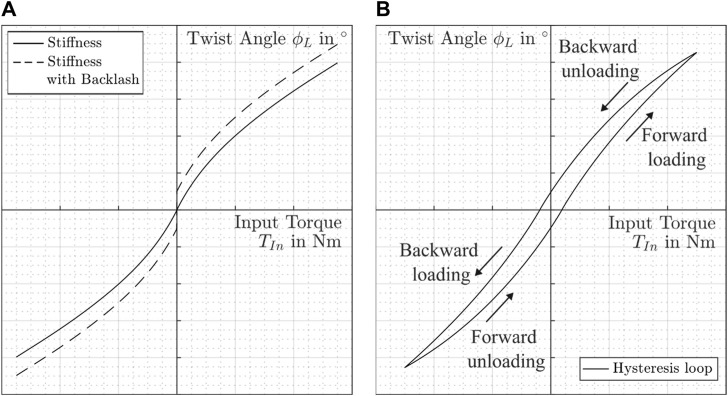
Example for the stiffness behavior. **(A)** Local compliance curve. **(B)** Hysteresis loop.

The stiffness can be evaluated with dependency on the applied torque. Therefore, the local compliance diagram is shown in Figure 4A for a case with backlash and a case without backlash.

The stiffness increases with rising torque and shows a nonlinear behavior. In combination with nonlinear friction, this leads to the hysteresis properties of gears for RLS (Mesmer et al., 2022). There are two possibilities to describe such a behavior experimentally. According to Dhaouadi et al. (2003) the output shaft of the gearbox is fixed, the input shaft is twisted in positive and negative direction in a sinusoidal manner, and the corresponding torque is recorded during the test. The second method is exactly the opposite (Mayr, 1989). Here, the input shaft is fixed and the gearbox output shaft is twisted. Most manufacturers use the second method to determine the stiffness behavior of their gearboxes. A typical example curve is shown in Figure 4B.

Gear manufacturer for RLS usually divide the compliance curve into three sections with constant stiffness values as a simple model for the nonlinearity. The torque limits of the individual ranges depend on the specific gearbox. A typical range for the value according to the maximum transmittable torque is given in Table 1.

**TABLE 1:** Definition of the stiffness values.

Symbol	Definition	Approximate range acc. T_max_
c_1_	Stiffness at low torque	∼0–10% of max. Torque
c_2_	Stiffness at middle torque	∼10–30% of max. Torque
c_3_	Stiffness at high torque	∼30–100% of max. Torque

The calculation models listed for stiffness and efficiency show differences in the level of detail. Therefore, a type-independent comparison of the different gearbox concepts is challenging. This paper aims to show different influence factors to the most relevant properties for gears of RLS independent of the specified gearbox type.

## 3 Main influence factors on selected properties

In this chapter, different influence factors on the two most relevant properties of robot gears are shown. The evaluation is based on manufacturer specifications for about 1,000 different gearboxes from various transmission manufacturers (Harmonic Drive AG, 2014a; Harmonic Drive AG, 2014b; Harmonic Drive AG, 2014c; Harmonic Drive AG, 2014c; Harmonic Drive AG, 2016a; Harmonic Drive AG, 2016b; Harmonic Drive AG, 2016c; Harmonic Drive AG, 2018; Harmonic Drive AG, 2019; Nabtesco, 2019; Sumitomo Drive Technologies, 2021; Neugart, 2023; Bonfiglioli, 2024). The strain wave drive (SW), planetary gear drive (PG) and cycloidal drive (CY) as the three main types of gears for robots are considered in the evaluation. Only precision gears with a low backlash were selected and are therefore suitable for precise drives such as those required by RLS. The selected precision gears cover a wide range of applications from small to large robots and RLS. The relevant influences on the two properties efficiency and stiffness are summarized in the following section. Detailed data is not available on all influences for the gearboxes under consideration. For this reason, the largest possible database for each gearbox type is used for the individual aspects.

### 3.1 Efficiency

In the gearboxes presented in this paper, the speed is transmitted exactly according to the transmission ratio. However, the torque and thus the power is subject to losses and is therefore not transmitted exactly according to the transmission ratio. The efficiency of a gearbox can be evaluated by the measured torque loss or the power loss. The influence of the gearbox size on these two values is of great significance. Since not all gearboxes have the same design structure, the influence was normalized to a relative size of the gearboxes in order to compare different gearboxes. This relative size is based on the manufacturer’s specification of the outer connection diameter closest to the ring gear and therefore has some information about the size of the gears inside the housing. The largest evaluated gearbox serves as a reference for the relative sizes and marks the 100% in the Figure.

**FIGURE 5:**
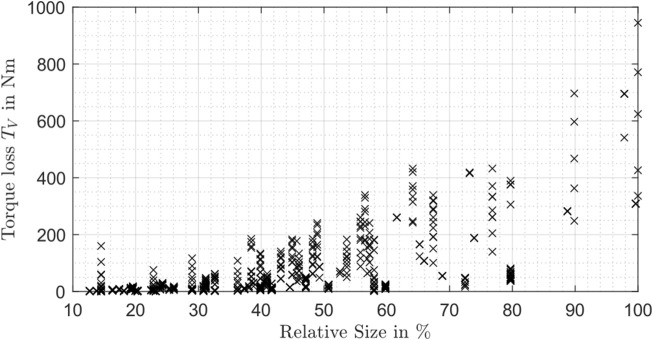
Torque loss influenced by the relative size for all evaluated gearboxes.

The influence of the gearbox size on the torque loss *T*_*V*_ at nominal conditions (individual nominal torque, nominal speed of 3,500 rpm) is shown in Figure 5 for all evaluated gearboxes (independent of the gearbox type). For small gearboxes the torque loss achieves values about 100 Nm, for large gearboxes the values go up to 1,000 Nm. There is a nonlinear, upward trend for the torque loss as the relative size of the gearbox increases. This is related to the fact that the torque to be transmitted from the input increases to a similar extent as the size of the gearbox increases.

In order to obtain a dimensionless parameter that is independent of the input power, the efficiency is calculated by the quotient of the output power to the input power (according to Equation 1). This efficiency value is dependent on the applied load for each individual gearbox. The data for all different gearbox types are analyzed at a speed of 3,500 rpm and an ambient temperature of 30°C for different torque levels to show this influence. For a size-independent representation, the torque values are standardized to the corresponding nominal torque.

**FIGURE 6:**
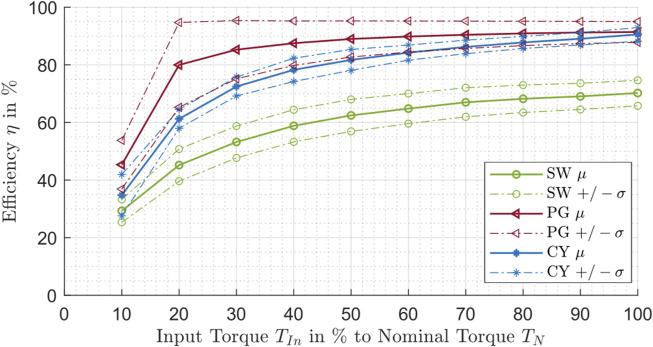
Efficiency influenced by the torque for the strain wave drive (SW), planetary gear drive (PG) and cycloidal drive (CY).

The arithmetic means and the standard deviations are calculated for different torque steps for the three drive types. The obtained curves for the mean values (marked with μ) and the standard deviations (marked with ±σ) are presented in Figure 6.

For all gearbox types, the efficiency increases with greater relative torque. The trends show similarities to a bounded growth behavior. The efficiency for planetary gear drives and cycloidal drives is especially in the low torque range increasing rapidly. The Figure also states that the evaluated planetary gear drives and the cycloidal drives have a generally higher efficiency than the strain wave drives.

**FIGURE 7:**
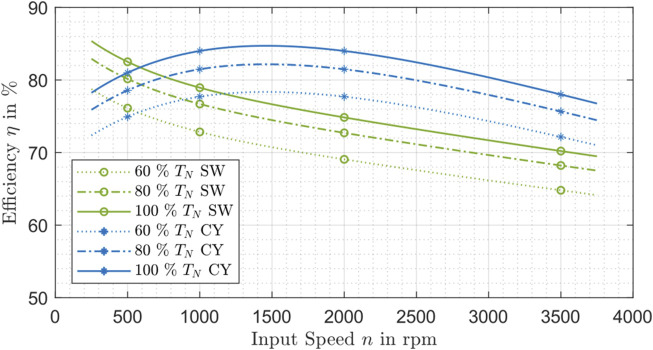
Efficiency influenced by the speed and torque for strain wave drive (SW) and cycloidal drive (CY).

In Figure 7 the influence of speed on the efficiency is evaluated and shown for strain wave drives and cycloidal drives. With the available manufacturer data, it is not possible to identify a dependency of the efficiency on the speed for planetary gearboxes. Therefore, the statistical mean of the efficiency is built in dependency on the speed at an ambient temperature of 30°C. This procedure is repeated for different torque levels relative to the according nominal torque.

The influence of speed on the efficiency of strain wave drives indicates a descending trend towards higher speeds. In contrary, the efficiency of cycloidal drives increases in the beginning and then also falls. In addition, the torque influence can be detected for both gear types with similar trend as mentioned and demonstrated in Figure 6.

**FIGURE 8:**
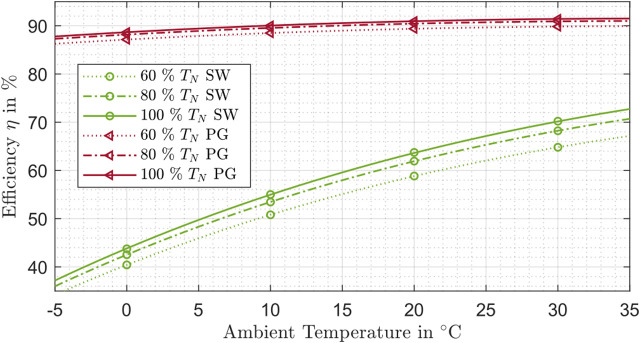
Efficiency influenced by the ambient temperature and torque for strain wave drive (SW) and planetary gear drive (PG).

This finding is also supported by Figure 8, which shows the influence of the ambient temperature and torque on the efficiency for different strain wave and planetary gear drives at a speed of 3,500 rpm. With the available manufacturer data, it is not possible to identify a dependency of the efficiency on the temperature for cycloidal drives.

The curves obtained show a positive trend for higher temperatures. Planetary gear drives show a flatter influence of the ambient temperature than the strain wave drives. In addition, the lower torque dependency of planetary gear drives can be seen. The efficiency values of the planetary gear drives are significantly higher in all areas than for corresponding strain wave drives (see also Figure 6).

**FIGURE 9:**
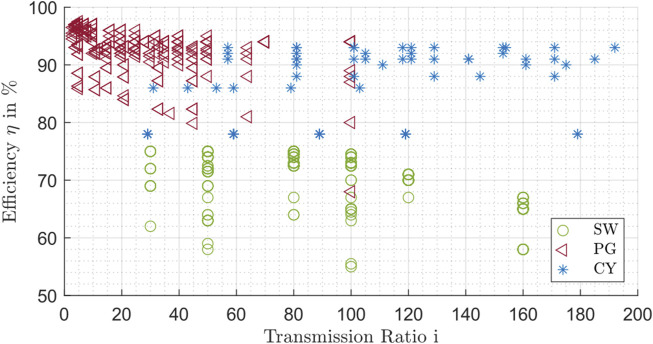
Efficiency influenced by the transmission ratio for planetary gear drives (PG), strain wave drives (SW) and cycloidal drive (CY).

In Figure 9, the influence of the transmission ratio on the efficiency at nominal conditions is evaluated for all gear drives.

The overall efficiency at nominal conditions is shown for the three types of drives in Figure 9. The different gearbox types show different ranges of transmission ratios. The planetary gears focus more on transmission ratios between 3 up to 50, whereas the strain wave and cycloidal drives are more represented in the transmission ratio range between 30 and 180. In addition, a drop in efficiency of the planetary gear drives can be detected at a gear ratio over approx. 10. One possible reason for this is the mechanical structure of the planetary gears. In order to achieve larger gear ratios, an additional gear stage is necessary, which in turn causes additional losses. A similar trend can be detected for the strain wave drives from a transmission ratio over 100. For the evaluated cycloidal drives, the efficiencies remain at approximately the same level, regardless of the transmission ratio. In addition, planetary gears and cycloid drives generally show higher efficiency than comparable strain wave drive. This confirms the statements of Figure 6.

### 3.2 Stiffness

To evaluate the influence on the stiffness of gears for RLS, the stiffness values from manufacturer specifications are chosen as data basis. As mentioned in Section 2.2, the manufacturers specify the stiffness in three sections in dependency on the applied torque. With the stiffness values *c*_*1*_, *c*_2_ and *c*_3_ (see Table 1) the non-linear load-deformation properties can be mapped (see Figure 4).

**FIGURE 10:**
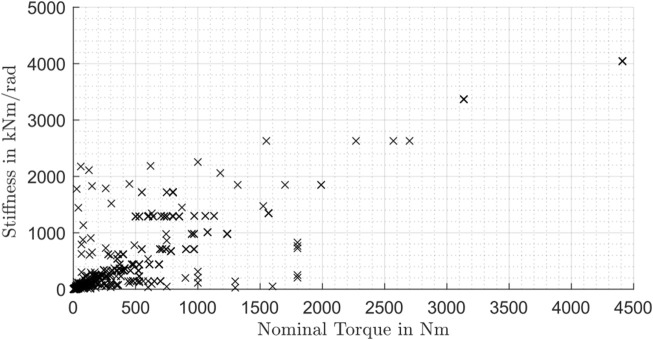
Stiffness influenced by the nominal torque for all types of gearboxes.

First of all, the influence of the nominal torque on the stiffness is of great interest. Therefore, the stiffness value *c*_3_ for the behavior at high torques is analyzed for all type of gearboxes in Figure 10.

**FIGURE 11:**
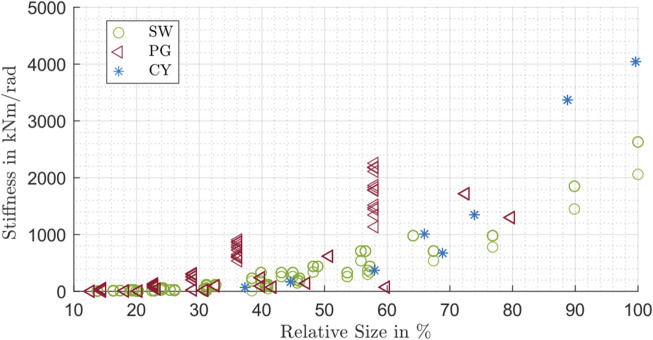
Stiffness *c*_3_ influenced by the relative size for strain wave drive (SW), planetary gear drive (PG) and cycloidal drive (CY).

The data in Figure 10 suggest a linear upward trend of the stiffness *c*_3_ with rising nominal torque. A possible reason for this is the relationship between the nominal torque and the gearbox size. If the transmittable nominal torque increases, usually the gearbox size and therefore also the stiffness values increase. In order to support this statement, the influence of the gearbox size is investigated in the next Figure for all gearbox types. The same approach to calculate the relative size was used as mentioned in Section 3.1. Figure 11 shows the influence of the relative size on the overall stiffness value:

The gearbox size shows a nonlinear upward trend to the stiffness behavior of the gears. This supports the general statement of Figure 10, however, the data confirms a trend similar to a quadratic or exponential dependency. For the different gearbox types shown in Figure 11, areas can be identified in which the respective type shows a relatively high stiffness behavior.

The same procedure can be applied on the stiffness values *c*_1_ and *c*_2_. With the available manufacturer data, it is only possible to consider the strain wave drives in a representative manner.

**FIGURE 12:**
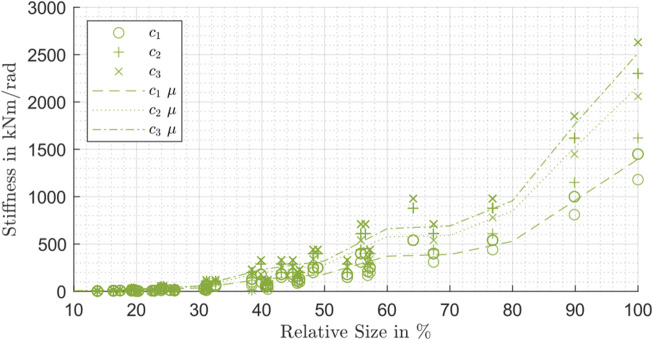
Stiffness *c*_1_, *c*_2_ and *c*_3_ influenced by the relative size for strain wave drive (SW).

In Figure 12, the three stiffness values of the strain wave drives are compared in dependency on the relative size. For a better visualization of the trends, the curves of the mean values (labeled as μ in Figure 12) are plotted as well. To get these mean values, the data was divided in nine groups dependent on the relative size. The stiffness values for the lower and middle torque range show the same behavior like the values of the higher torque range and increase with rising relative size. The mean curves of *c*_2_ and *c*_3_ have almost a similar slope. Generally, the values of *c*_2_ are closer to the values of *c*_3_ than to *c*_1_.

With the available manufacturer data, it is not possible to identify an influence of the speed, ambient temperature and the transmission ratio on the stiffness behavior.

## 4 Conclusion

This paper gives an overview of relevant properties of gears for robots and RLS (see Figure 2). Efficiency and stiffness as the two most important properties are analyzed in more detail. Therefore, the influences of different parameters on these two properties are shown. In summary, this analysis provides insights into the factors affecting the efficiency and stiffness of planetary gear, cycloidal and strain wave drives, including size, torque, speed, ambient temperature and transmission ratio.

The following conclusions can be drawn from the investigation.

- For all of the three evaluated types, the increasing size of the gearboxes leads to an increase in stiffness.
- The torque and speed of the gearboxes mainly influence the efficiency behavior of the drives. Especially in low torque ranges, the efficiency of the evaluated planetary gearboxes is higher than that of the other two drives.
- The ambient temperature only has a significant influence on the efficiency. At higher temperatures, the efficiency increases.
- The influence of the transmission ratio does not show a clear trend either in the efficiency or in the stiffness.

In a further research additional factors like material behavior (e.g., replacing steel with plastic) or the manufacturing process can be investigated.

## Data Availability

The original contributions presented in the study are included in the article/supplementary material, further inquiries can be directed to the corresponding author.
